# Pregnancy Intention, Changes in Pregnancy Intention, and Pregnancy Incidence Among Female Nurses in North America

**DOI:** 10.1001/jamanetworkopen.2023.11301

**Published:** 2023-05-03

**Authors:** Siwen Wang, Lidia Minguez-Alarcon, Melissa Perez Capotosto, Makiko Mitsunami, Audrey J. Gaskins, Brittany M. Charlton, Jaime E. Hart, Janet W. Rich-Edwards, Jorge E. Chavarro

**Affiliations:** 1 Department of Nutrition, Harvard T.H. Chan School of Public Health, Boston, Massachusetts; 2 Channing Division of Network Medicine, Department of Medicine, Brigham and Women’s Hospital and Harvard Medical School, Boston, Massachusetts; 3 Connell School of Nursing, Boston College, Boston, Massachusetts; 4 Department of Epidemiology, Rollins School of Public Health, Emory University, Atlanta, Georgia; 5 Department of Population Medicine, Harvard Medical School and Harvard Pilgrim Health Care Institute, Boston, Massachusetts; 6 Department of Epidemiology, Harvard T.H. Chan School of Public Health, Boston, Massachusetts; 7 Department of Environmental Health, Harvard T.H. Chan School of Public Health, Boston, Massachusetts; 8 Division of Women’s Health, Department of Medicine, Brigham and Women’s Hospital, Harvard Medical School, Boston, Massachusetts

## Abstract

**Importance:**

Pregnancy intention assessment is a key element of preconception and contraceptive care. The association between a single screening question and the incidence of pregnancy is unknown.

**Objective:**

To prospectively evaluate the dynamics of pregnancy intention and pregnancy incidence.

**Design, Setting, and Participants:**

This prospective cohort study (the Nurses’ Health Study 3) was conducted from June 1, 2010, to April 1, 2022, in 18 376 premenopausal, nonpregnant female nurses aged 19 to 44 years.

**Main Outcomes and Measures:**

Pregnancy intention and pregnancy status were assessed at baseline and approximately every 3 to 6 months thereafter. Cox proportional hazards regression models were used to estimate the association between pregnancy intention and pregnancy incidence.

**Results:**

A total of 18 376 premenopausal, nonpregnant women (mean [SD] age, 32.4 [6.5] years) participated in the study. At baseline, 1008 women (5.5%) were trying to conceive, 2452 (13.3%) were contemplating pregnancy within 1 year, and the remaining 14 916 (81.2%) were neither trying to conceive nor thought they would be pregnant within 1 year. A total of 1314 pregnancies were documented within 12 months of pregnancy intention assessment. The cumulative incidence of pregnancy was 38.8% in women actively trying to conceive (median [IQR] time to pregnancy, 3.3 [1.5-6.7] months), 27.6% in women contemplating pregnancy (median [IQR] time to pregnancy, 6.7 [4.2-9.3] months), and 1.7% in women neither trying to conceive nor contemplating pregnancy (median [IQR] time to pregnancy, 7.8 [5.2-10.5] months) among those who became pregnant. Women who were actively trying to conceive were 23.1 times (95% CI, 19.5-27.4 times) and women who were contemplating pregnancy were 13.0 times (95% CI, 11.1-15.2 times) more likely to conceive within 12 months than women who were neither attempting nor contemplating pregnancy. Among women contemplating pregnancy at baseline who did not get pregnant during follow up, 18.8% were actively trying and 27.6% were not trying by 12 months. Conversely, only 4.9% of women neither trying to conceive nor contemplating pregnancy within 1 year at baseline changed pregnancy intention during follow up.

**Conclusions and Relevance:**

In this cohort study of reproductive-aged nurses in North America, pregnancy intention was highly fluid among women who were contemplating pregnancy but relatively stable among women trying to conceive and women who were neither trying to conceive nor contemplating pregnancy. Pregnancy intention was strongly associated with pregnancy incidence, but the median time to pregnancy points to a relatively short time window to initiate preconception care.

## Key Points

**Question:**

Is stated pregnancy intention stable and associated with incidence of pregnancy among nurses of reproductive age?

**Findings:**

In this prospective cohort study of 18 376 premenopausal, nonpregnant, female nurses, those who were actively trying to become pregnant were 23 times more likely to conceive within 12 months, and those contemplating pregnancy were 13 times more likely to conceive within 12 months compared with women who were neither attempting to become pregnant nor contemplating pregnancy. Approximately half (46%) of women who were contemplating pregnancy at baseline changed pregnancy intention during follow-up.

**Meaning:**

These findings suggest that preconception pregnancy intention is fluid but strongly associated with pregnancy incidence.

## Teaser

This cohort study of reproductive-age nurses evaluates the association of pregnancy intention and changes in pregnancy intention with the probability of pregnancy during a 12-month period.

## Introduction

The World Health Organization and the American College of Obstetricians and Gynecologists recommend implementing preconception counseling in primary care.^1,2^ However, this recommendation relies on the ability of practitioners to identify those who are likely to become pregnant. Evidence indicates that pregnancy intention is not widely assessed among women of reproductive age.^3,4,5^ Nationally representative data suggest that only 44% of women presenting to primary care receive reproductive health care,^5^ and more than half of pregnancies are estimated to be unintended.^6^ Furthermore, despite being recommended to all women of childbearing age in the US, only 1 in 4 nonpregnant women were regularly taking folic acid supplements before pregnancy, with a similar percentage of women reporting receiving this recommendation from health care practitioners.^3,4^ Some women may also change their intention or report it differently because of social desirability.^7,8,9,10,11^ Moreover, conventional measures of a binary intended vs unintended pregnancy may not adequately capture the true spectrum of differences in attitudinal dimensions of childbearing desire.^12^

These situations underscore the need for a more nuanced approach to assess pregnancy intention. The One Key Question initiative recommends that primary care practitioners screen nonpregnant women with the question, “Would you like to become pregnant in the next year?”^13^ However, its validation is limited to psychometric properties,^14^ and it is unclear to what extent this or similar questions predict the future probability of pregnancy. Therefore, although intuitively appealing, the clinical utility of such a question has never been studied. In this study, we used data from a longitudinal cohort to evaluate how a pregnancy intention screening question is associated with the probability of pregnancy during a 12-month period and the changes in pregnancy intention within women over time.

## Methods

### Study Design and Population

Participants were drawn from an ongoing web-based open cohort, the Nurses’ Health Study 3 (NHS3). Launched in June 2010, the NHS3 includes female and male registered nurses or nursing students in the US and Canada born on or after January 1, 1965.^15^ As of April 2022, a total of 48 919 women had joined the study. Approximately every 6 months, follow-up questionnaires are sent to participants to update lifestyle, reproductive, and medical information. Additional questionnaires are sent every 3 months to participants who report being actively trying to get pregnant, querying on their pregnancy and pregnancy intention status (eFigure 1 in Supplement 1).

Because follow-up questionnaires may not be returned exactly at 6-month intervals and because some women may not be aware of pregnancy during the first few weeks, we followed up participants for 18 months to ascertain pregnancies that occurred within 12 months of completion of the baseline questionnaire. The end of follow-up of the current study was April 1, 2022, and participants must have completed their baseline questionnaire before October 1, 2020 (N = 48 700).

**Figure 1.**
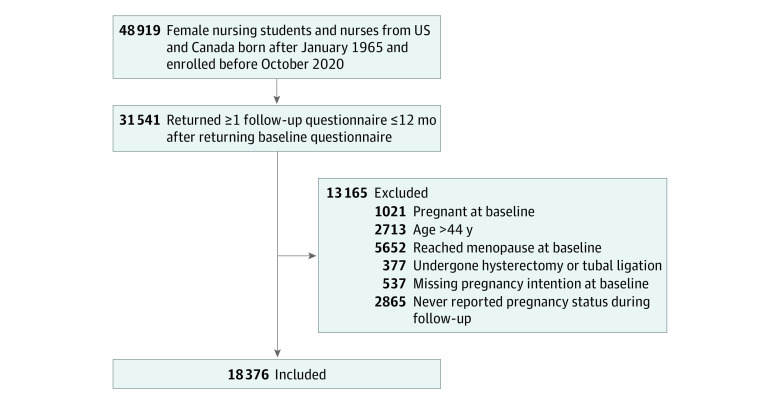
Flowchart of Selection of the Study Participants, Nurses’ Health Study 3 (2010-2022) A total of 2519 individuals lost to follow-up were censored at last return of the questionnaire.

Participants were eligible for the current study if they had returned at least 1 follow-up questionnaire within 18 months after the baseline questionnaire (n = 31 541). We excluded women who, at baseline, were pregnant (n = 1021), older than 44 years (n = 2713), had reached menopause or were uncertain about menopausal status (n = 5653), or had undergone hysterectomy (n = 59) or tubal ligation (n = 318). We also excluded participants who did not report their baseline pregnancy intention status (n = 537) and women whose pregnancy status was not reported during follow-up (n = 2865), leaving 18 376 women in the analysis (Figure 1). Women included in the final analytical sample were more likely to be younger, non-Hispanic White, and nulliparous than women excluded from analysis, but no other differences were identified between these groups (eTable 1 in Supplement 1). The study protocol was approved by the institutional review board of the Brigham and Women’s Hospital. Return of questionnaires implied informed consent. This study followed the Strengthening the Reporting of Observational Studies in Epidemiology (STROBE) reporting guideline for cohort studies.

### Pregnancy Status and Pregnancy Intention

Pregnancy intention and pregnancy status were prospectively ascertained using a series of logically nested questions. Women were first asked if they were currently pregnant. Women who were not pregnant were then asked, “Are you actively trying to become pregnant, or do you think that you may become pregnant at some point within the next year?” Response options included “No,” “Yes, actively trying,” and “Yes, may become pregnant within the next year” (termed as *contemplating pregnancy* hereafter). If actively trying, women were asked about how many months they have been trying and whether they had stopped using contraception. These questions were offered on the baseline questionnaire and in each follow-up questionnaire (eFigure 1 in Supplement 1). We plotted the distribution of the current duration of pregnancy attempt (in months) among women who ever reported being actively trying to get pregnant and compared it with the expected log-normal distribution to account for the length-based sampling.^16^ The observed distribution was comparable to the expected distribution (eFigure 2 in Supplement 1).

### Covariates

Demographic, reproductive, and socioeconomic information was self-reported at baseline, including age, race and ethnicity, residential address, marital status, pregnancy history, and educational attainment of the participant and, if partnered, of their partner. We included race and ethnicity information because of known difference in pregnancy intendedness by racial and ethnic groups. Current contraceptive use was queried during follow-up. Household income was collected in a subsample of 11 361 women (61.8%). Information on religiosity was collected among 10 470 women (57.0%) who returned a supplemental questionnaire.

### Statistical Analysis

Individuals were followed up from return of baseline questionnaire until pregnancy or 12 months after the date of completion of the baseline questionnaire, whichever came first. Participants lost to follow-up (n = 2519 [13.7%]) were censored at return of last questionnaire. Cumulative incidence of pregnancy was estimated by the Kaplan-Meier method using time in study as the time scale; differences between groups was assessed using the log-rank test.

We used proportional hazards regression to estimate the hazard ratios (HRs) and 95% CIs of the associations of baseline and time-varying pregnancy intention with pregnancy incidence during follow-up, adjusting for age, race and ethnicity, marital status, and educational attainment. We conducted stratified analysis by women’s demographic and socioeconomic characteristics that are strongly related to pregnancy intention.^6,12,17^ Interaction effects were assessed using cross-product terms. Missingness of each variable was less than 5%. Indicator variables were used for any missing covariate information for categorical variables. Missing values of continuous variables were replaced with the median.^18^

Finally, among those who reported pregnancy intention status twice or more and did not get pregnant during follow-up, we examined changes in pregnancy intention (n = 8449). For this analysis, participants were followed up until the date when they last reported pregnancy intention during the study period.

We conducted several sensitivity analyses. First, because contraceptive use was not comprehensively measured at baseline, we examined the cross-sectional correlation between pregnancy intention and current contraception use in a follow-up questionnaire when these 2 questions were simultaneously queried. Second, because of concerns about age-related fertility decline, we restricted analyses to women younger than 35 years (n = 11 288). Third, to reduce the potential impact of the COVID-19 pandemic on pregnancy intention, we ended follow-up on December 31, 2019 (n = 18 003). Fourth, we excluded participants who were younger than 22 years (n = 749), were not partnered (n = 7151), were using contraception (n = 5, only queried in actively trying group), had a history of infertility treatment (n = 28), or had been trying for 6 months or more (n = 533) at baseline to mirror the inclusion criteria into preconception studies.^19,20,21^ Fifth, because only participants who reported trying to get pregnant were asked about pregnancy intention quarterly, we restricted pregnancy intention analyses to reports on main cohort questionnaires only. All analyses were performed with SAS software, version 9.3 for UNIX (SAS Institute Inc). All statistical tests were 2-sided, and *P* < .05 was considered statistically significant.

## Results

A total of 18 376 premenopausal, nonpregnant women (mean [SD] age, 32.4 [6.5] years) participated in the study. Most participants were born (16 978 [92.4%]) and resided (17 552 [95.5%]) in the US. At baseline, 1008 women (5.5%) were actively trying to conceive, 2452 (13.3%) were contemplating pregnancy, and the remaining 14 916 (81.2%) were neither trying to conceive nor contemplating pregnancy. In the subgroup of women asked about contraception use at the same time as pregnancy intention, 320 women (93.6%) reporting being actively trying were not using contraception, whereas 500 women contemplating pregnancy (68.6%) and 4037 women not trying to conceive (73.6%) were using contraception (eTable 2 in Supplement 1). Nearly 1 in 3 women (33.7%) actively trying to conceive at baseline had been trying for 12 months or more. Women actively trying to conceive were more likely to be married or in a domestic relationship, be nulliparous, and attend religious services more often than women in the other 2 groups (Table 1).

**Table 1.** Baseline Sociodemographic Characteristics According to Prospectively Reported Preconception Pregnancy Intention at Baseline in 18 376 Women, Nurses’ Health Study 3 (2010-2022)^a^

Characteristic	Actively trying (n = 1008 [5.5%])	Contemplating pregnancy within 1 y (n = 2452 [13.3%])	Neither trying nor thinking will become pregnant (n = 14 916 [81.2%])
Time actively trying, mean (SD), mo.	7.6 (5.1)	NA	NA
Currently using contraception^b^	9 (0.9)	NA	NA
Trying for more than 12 mo^b^	340 (33.7)	NA	NA
Age, mean (SD), y	32.5 (4.6)	31.0 (4.5)	32.6 (6.8)
Age groups, y			
19-24	23 (2.3)	123 (5.0)	2127 (14.3)
25-34	662 (65.7)	1783 (72.7)	6570 (44.1)
35-44	323 (32.0)	546 (22.3)	6219 (41.7)
Race and ethnicity			
Non-Hispanic White	863 (85.6)	2180 (88.9)	13 128 (88.1)
Hispanic	56 (5.6)	92 (3.8)	617 (4.1)
All other minority groups^c^	89 (8.8)	180 (7.3)	1171 (7.9)
Marital status			
Never married	49 (4.9)	375 (15.3)	6147 (41.2)
Married or domestic partnership	931 (92.4)	1982 (80.8)	7609 (51.0)
Separated or divorced	25 (2.5)	93 (3.8)	1094 (7.3)
Widowed	0	1 (.04)	27 (0.2)
Nulliparous	679 (67.4)	1498 (61.1)	8344 (55.9)
Educational attainment			
Diploma in nursing	32 (3.2)	66 (2.7)	500 (3.4)
Associate’s degree	507 (50.3)	1330 (54.2)	7788 (52.2)
Bachelor’s degree	245 (24.3)	540 (22.0)	2595 (17.4)
Master’s degree or doctorate	15 (1.5)	33 (1.4)	229 (1.5)
Partner’s educational attainment^d^			
High school or lower	348 (37.4)	642 (32.4)	3008 (39.5)
College	337 (36.2)	837 (42.2)	2852 (37.5)
Graduate school	246 (26.4)	502 (25.3)	1740 (22.9)
Region			
West	208 (20.6)	496 (20.2)	2906 (19.5)
Midwest	300 (29.8)	718 (29.3)	4220 (28.3)
South	235 (23.3)	537 (21.9)	3340 (22.4)
Northeast	211 (20.9)	566 (23.1)	3686 (24.7)
Military or outside US	54 (5.4)	135 (5.5)	764 (5.1)
Household income, $			
<50 000	24/597 (4.0)	86/1473 (5.8)	1074/9291 (11.6)
50 000-99 999	142/597 (23.8)	390/1473 (26.5)	2699/9291 (29.1)
100 000-199 999	314/597 (52.6)	748/1473 (50.8)	4147/9291 (44.6)
>200 000	85/597 (14.2)	197/1473 (13.4)	1050/9291 (11.3)
Religiosity			
Attended religious services once a week or more	132/536 (24.6)	274/1337 (20.5)	1699/8597 (19.8)
Participated in private religious activity more than once a week	198/536 (36.9)	484/1337 (36.2)	2896/8597 (33.7)

Abbreviation: NA, not applicable.

^a^ Data are presented as number (percentage) of women unless otherwise indicated. Numbers might not total 100% because of missingness.

^b^ Only queried among women who reported being actively trying.

^c^ African American or Black, American Indian or Alaska Native, Asian or Native Hawaiian or Pacific Islander (groups combined because the numbers of subgroups are too small).

^d^ Unpartnered women were not included.

**Figure 2.**
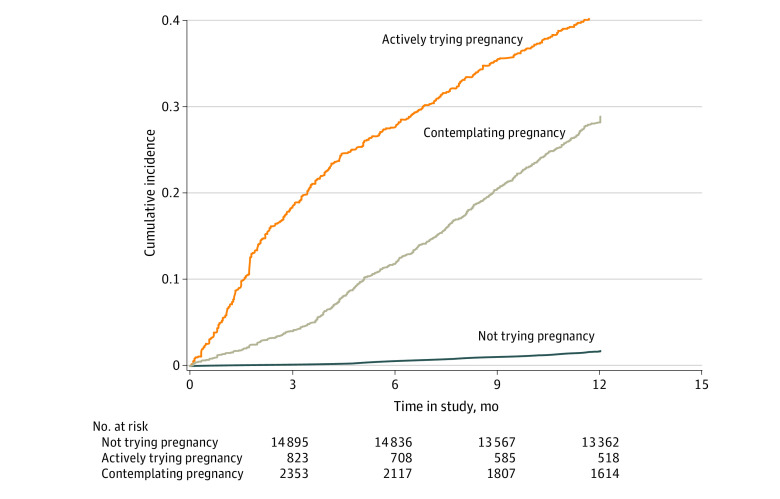
Kaplan-Meier Estimates of Cumulative Pregnancy Incidence Within 12 Months According to Prospectively Reported Preconception Pregnancy Intention at Baseline, Nurses’ Health Study 3 (2010-2022)

We documented 1314 pregnancies within 12 months of baseline pregnancy intention assessment. Women who were actively trying to get pregnant at baseline were more likely to become pregnant at any point during follow-up than women in the other 2 groups (Figure 2). The crude cumulative probabilities of pregnancy at 12 months were 38.8% (391 of 1008) in women actively trying to conceive, 27.6% (677 of 2452) in women contemplating pregnancy, and 1.7% (246 of 14 916) in those neither trying to conceive nor contemplating pregnancy (Table 2). Compared with women who were neither trying to conceive nor contemplating pregnancy, women trying to conceive were 23.1 (95% CI, 19.5-27.4) times more likely and women contemplating pregnancy were 13.0 (95% CI = 11.1-15.2) times more likely to conceive within 12 months, after adjusting for demographic factors. Among women who became pregnant during follow-up, the time between pregnancy intention assessment and pregnancy also differed among these 3 groups, with a median (IQR) of 3.3 (1.5-6.7) months in women actively trying to conceive, 6.7 (4.2-9.3) months in women contemplating pregnancy, and 7.8 (5.2-10.5) months in women neither trying to conceive nor contemplating pregnancy (Table 2). Associations were stronger when using time-varying pregnancy intention as the independent variable (Table 2). The associations between baseline pregnancy intention and pregnancy within 12 months differed by age, partnership status, race and ethnicity, and parity but not by educational attainment (Table 3). Pregnancy intention status was more strongly associated with pregnancy within 12 months among women who were 35 years or older, non-Hispanic White, unpartnered, or nulliparous. Cumulative risks of pregnancy were higher among women younger than 35 years and when excluding women with a history of infertility and women who had been trying for 6 or more cycles at baseline (eFigures 3 and 4 in Supplement 1). Results were similar when we excluded follow-up during the COVID-19 pandemic (eFigure 5 in Supplement 1).

**Table 2.** HRs (95% CIs) of Pregnancy Within 12 Months According to Prospectively Reported Preconception Pregnancy Intention in 18 376 Women, Nurses’ Health Study 3

Baseline pregnancy intention	No. of women	No. pregnant within 12 mo	Incidence of pregnancy, %	Time to pregnancy from study entry, median (IQR), mo^b^	HR (95% CI)		
					Age-adjusted, baseline intention only	Multivariable adjusted^a^	
						Baseline intention only	Time-varying intention^c^
All nonpregnant women	18 376	1314	7.2	6.0 (3.3-9.0)	NA	NA	NA
Actively trying	1008	391	38.8	3.3 (1.5-6.7)	34.8 (29.6-40.9)	23.1 (19.5-27.4)	31.2 (26.3-37.1)
Contemplating	2452	677	27.6	6.7 (4.2-9.3)	18.5 (16.0-21.4)	13.0 (11.1-15.2)	14.2 (12.0-16.7)
Neither trying nor thinking will become pregnant	14 916	246	1.7	7.8 (5.2-10.5)	1 [Reference]	1 [Reference]	1 [Reference]

Abbreviations: HR, hazard ratio; NA, not applicable.

^a^ Adjusted for age (continuous), educational attainment (diploma in nursing/associate’s degree, bachelor’s degree, master’s degree or doctorate, or missing), marital status (married or domestic partner, unpartnered, or missing), and race (non-Hispanic White, Hispanic, or other [African American or Black, American Indian or Alaska Native, Asian or Native Hawaiian or Pacific Islander]).

^b^ Among women who became pregnant during follow-up.

^c^ Sample size is 8449 who completed at least 2 pregnancy intention questionnaires.

**Table 3.** HRs (95% CIs) for Pregnant Within 12 Months in 18 376 Women According to Preconception Pregnancy Intention at Baseline, Stratified by Socioeconomic Factors, Nurses’ Health Study 3

Characteristic	No. of pregnancies/No. of women	Adjusted HR (95% CI)^a^			*P* value for interaction
		Actively trying	Contemplating pregnancy	Neither	
Age group, y					
18-24	46/2273	30.4 (11.14-82.73)	12.8 (5.9-27.6)	1 [Reference]	<.001
25-34	1053/9015	16.2 (13.3-19.6)	9.1 (7.6-10.8)	1 [Reference]	
35-44	215/7088	37.5 (25.6-55.0)	28.80 (20.0-41.4)	1 [Reference]	
Race and ethnicity					
Non-Hispanic White	1175/16 171	24.4 (20.4-29.3)	13.4 (11.3-15.8)	1 [Reference]	.09
Hispanic, any race	52/765	17.2 (7.3-40.8)	15.9 (7.2-35.3)	1 [Reference]	
All other minority groups	87/1440	14.5 (7.9-26.8)	8.2 (4.7-14.4)	1 [Reference]	
Marital status					
Unpartnered	155/7854	33.8 (19.0-60.0)	20.2 (19.0-28.4)	1 [Reference]	<.001
Married or domestic partner	1159/10 522	19.4 (16.2-23.2)	10.5 (8.8-12.5)	1 [Reference]	
Educational attainment					
Diploma in nursing or associate’s degree	41/598	11.4 (4.5-28.7)	10.9 (4.9-23.9)	1 [Reference]	.31
Bachelor’s degree	731/9625	23.0 (18.3-30.0)	12.4 (10.0-15.2)	1 [Reference]	
Master’s degree or doctorate	312/3657	26.3 (18.1-38.2)	14.9 (10.5-21.2)	1 [Reference]	
Parity					
Nulliparous	761/10 521	25.3 (19.6-32.6)	14.4 (11.4-18.1)	1 [Reference]	.09
Parous	553/7855	18.6 (14.5-23.8)	9.29 (7.4-11.6)	1 [Reference]	

Abbreviation: HR, hazard ratio.

^a^ Adjusted for age (continuous), educational attainment (diploma in nursing/associate’s degree, bachelor’s degree, master’s degree or doctorate, or missing), marital status (married or domestic partner, unpartnered, or missing), and race and ethnicity (non-Hispanic White, Hispanic, or other [African American or Black, American Indian or Alaska Native, Asian or Native Hawaiian or Pacific Islander]), except for the stratifying variable.

A total of 8449 of the 17 062 women (49.5%) who completed a baseline pregnancy intention assessment also completed 1 or more follow-up intention assessments; 949 (11.2%) of these participants changed their pregnancy intention status during follow-up. Most of the changes in intention status were among women who were contemplating pregnancy at baseline (n = 856), of whom 398 (46.5%) changed pregnancy intention by the end of follow-up: 161 (18.8%) reported their intention as actively trying, and 237 (27.7%) reported their intention as not trying (eFigure 6 in Supplement 1). The number of women changing their intention status during follow-up was 131 (24.4%) among 535 women who initially reported being actively trying and 344 (4.9%) among 7058 women initially reporting neither trying to conceive nor contemplating pregnancy. Overall, women were more likely to change their pregnancy intention if they were aged 25 to 34 years, married or in a domestic relationship, nulliparous, and had higher education attainment (eTables 3-6 in Supplement 1). The frequency of change in pregnancy intention was comparable (703 of 7958 [8.8%]) when only data from main follow-up questionnaires were used.

## Discussion

Results from this prospective cohort study of more than 18 000 nonpregnant women of reproductive age showed that pregnancy intention was associated with pregnancy within the subsequent 12 months. Overall, 38.8% of women who were actively trying to conceive and 27.6% of those contemplating pregnancy became pregnant within a year of answering the pregnancy intention question. The 12-month cumulative pregnancy rate among women reporting neither trying nor contemplating (1.7%) was close to contraceptive failure rates in the general population.^22^ Among participants reporting their intention twice or more, approximately half of women contemplating pregnancy at baseline changed their pregnancy intention within 12 months. The One Key Question was proposed as an opening question to start a conversation about women’s reproductive health needs and to prevent unintended pregnancies.^13,23^ Our findings suggest that routine screening for pregnancy intention may have direct clinical applicability by simultaneously identifying women in need of contraceptive services, preconception care, or referral to infertility services. More importantly, more frequent screening may be warranted to account for changes in pregnancy intention.

Our study provides estimated likelihood of pregnancy during 12 months and median time from study entry to pregnancy by 3 different pregnancy intentions. The cumulative incidence and median time to pregnancy from study entry of the actively trying group observed in our study were similar to that of a large (N = 2 224 068), nationally representative cohort study^24^ in China of couples attempting pregnancy (cumulative pregnancy rate, 42%; median [IQR] time to pregnancy, 3 [2-5] months). Another longitudinal study^25^ of 889 women observed that within 2 years of follow-up, a pregnancy occurred in 28% of women who were contemplating pregnancy at baseline and 5% of women who were not. The cumulative incidence of pregnancy during 12 months was lower than that in clinical trials recruiting women who were planning pregnancy (approximately 48%-69%).^19,20^ However, these studies preferentially enrolled participants based on characteristics likely to influence incidence of pregnancy, such as partnership status, age, and history of infertility. Nevertheless, the cumulative incidence findings are likely to be biased toward underestimating the probability of pregnancy within a year and need to be interpreted with caution. First, change in pregnancy intention during follow-up affected the estimate of months of actual pregnancy attempt. Second, women with longer duration of pregnancy attempt are more likely to stay in the actively trying group using the current duration approach.^26^ Third, pregnancy intentions and pregnancy behaviors are not exchangeable. Women who reported being actively trying to conceive may still be using contraceptives and thus not at risk for pregnancy, and vice versa, as shown in our study (eTable 2 in Supplement 1) and others.^27,28,29,30^ For instance, in a retrospective qualitative study,^27^ only half of women who held a positive preconception desire for pregnancy had discontinued contraception and were having unprotected intercourse. Regardless, these estimates have important implications for primary care practitioners. They show that the window of opportunity to initiate preconception interventions can be relatively narrow among women actively trying to conceive.

Family planning is influenced by societal, psychological, and biological factors that are not stable over time, such as marital status, career goals, and health conditions, among other life situations.^17,31,32,33,34,35^ In our study, during 12 months of follow-up, approximately 1 in 9 women changed their stated pregnancy intention. Change in pregnancy intention status was particularly common (46.5%) among women who may have been more ambivalent about pregnancy (contemplating), and this group leaned slightly toward avoiding pregnancy (18.8% changed to actively trying and 27.6% changed to not trying). This is a largely undercharacterized but important group between 2 extremes of a pregnancy intention continuum.^36^ As shown here and in several prior studies,^36,37,38^ if given this option, 13% to 40% women would classify themselves into this category. On the other hand, pregnancy intention among women who were avoiding pregnancy was fairly stable, in agreement with a previous study^35^ that found that women who reported never desiring a pregnancy had the most stable pregnancy intention (78% did not change) at 12-month reassessment, whereas 60% of those contemplating pregnancy within the next year changed their desire. Psychometric evaluation of the One Key Question also suggests that this question performs best as a measure of the intent to avoid pregnancy.^14^ However, the clinical consequences (eg, unintended pregnancies) of the change in pregnancy intention among those who originally stated that they did not desire a future pregnancy should not be overlooked, reinforcing the importance of frequent reassessment of pregnancy intention.

We found that pregnancy intention was less associated with pregnancy within 1 year among women who were in the 25- to 34-year age group, were partnered, were parous, or belonged to racial or ethnic minorities compared with their counterparts. This finding could be attributable to the greater fluidity among participants in these groups because pregnancy intentions are more strongly associated with fertility behaviors among those who hold intention with greater certainty.^39^ Notably, pregnancy intention is strongly associated with pregnancy among women 35 years or older, indicating that the association is not simply based on age-related fertility and the need to measure pregnancy intention regardless of demographic factors, marital status, and reproductive history.

The prevalence of 12-month infertility estimated by current duration method among the actively trying group in our study was 33.7%. This estimate is substantially higher than that in surveillance data in the US, which use a constructed approach known to underestimate the prevalence of infertility due to recall and selection bias, especially as some estimates are restricted to women who are partnered and pregnant.^26,40,41^ Notably, our study did not restrict the study sample based on sexual activity, contraception use, relationship status, or history of infertility in order to resemble the real-world implication of such screening questions. Our estimate for 12-month infertility is comparable to that previously reported in France using the same methodo (34%),^42^ and the observed distribution in our cohort matched the expected distribution (eFigure 3 in Supplement 1). Although a discussion of estimating the prevalence of infertility is beyond the scope of this article, our findings highlight how different methodological approaches to assess the frequency of infertility can yield vastly different estimates.^40^

### Strengths and Limitations

Our study is one of the largest prospective studies with frequent follow-up intervals at 3 to 6 months of pregnancy intention and pregnancy status among women of reproductive age. Other strengths of our study include the use of a single question, which is easy to incorporate into clinical settings. At the same time, our findings suggest that in research contexts, additional attention to the fluidity of pregnancy intention may be important. We were also able to account for the important social and demographic risk factors of unintended childbearing.

Our study has several limitations. First, all study participants were nurses, and most of them were non-Hispanic White and older than reproductive-aged women in the general US population, limiting generalizability of our findings. Nurses may have more access to information on contraception and fertility and greater adherence to contraceptive use, and some of their job characteristics may have an effect on family planning.^43,44^ Future studies are needed to evaluate whether our findings are reproducible in more diverse populations. Second, we did not have information about the pregnancy intention of participants’ partners, which is very influential on female pregnancy intention and pregnancy incidence.^33,39^ Third, although we were able to examine the overlap between pregnancy intention and contraceptive practices in a subgroup of women, we did not have contraceptive use at baseline except for those who were actively trying, which precludes us from investigating the interplay among contraceptive use, pregnancy intention, and pregnancy incidence in the entire study population.

## Conclusions

In this cohort study, we found that preconception pregnancy intention was fluid but strongly associated with pregnancy incidence. In addition, the observed median time to pregnancy suggested a relatively short time window to initiate preconception interventions among women who were actively trying to conceive. Our findings suggest that screening for pregnancy intention among reproductive-aged women may help identify women with high likelihood of becoming pregnant within the next year and would thus benefit from preconception counseling to improve population-level pregnancy outcomes. The development of appropriate strategies to evaluate pregnancy intention would benefit from a more nuanced understanding of the prospect of pregnancy.

## Notes

Supplement 1. **eFigure 1.** Flowchart of Cohort Design, Nurses’ Health Study 3 (NHS3), 2010–Present **eFigure 2.** Log-Normal Survival Function of the Duration of Ongoing Pregnancy Attempt (Solid Line) and Actual Distribution of Ongoing Pregnancy Attempt (Black Dots) Among Women Who Ever Reported Being Actively Trying to Get Pregnant, Nurses’ Health Study 3 Cohort (N = 5,249) **eFigure 3.** Kaplan-Meier Estimates of Cumulative Pregnancy Incidence Within 12 Months According to Prospectively Reported Preconception Pregnancy Intention at Baseline Among Women Aged 35 Years or Younger, Nurses’ Health Study 3 (2010–2022) **eFigure 4.** Kaplan-Meier Estimates of Cumulative Pregnancy Incidence Within 12 Months According to Prospectively Reported Preconception Pregnancy Intention at Baseline Among Partnered Women Aged 22 Years or Older, Did Not Report a History of Infertility, and Had Not Been Trying for Less Than 6 Months at Baseline, Nurses’ Health Study 3 (2010–2022) **eFigure 5.** Kaplan-Meier Estimates of Cumulative Pregnancy Incidence Within 12 Months According to Prospectively Reported Preconception Pregnancy Intention at Baseline, Restricting Follow up to Prior to the Pandemic, NHS3 (2010–2020) **eFigure 6.** Change in Pregnancy Intention Over 12 Months of Follow-up (Last Returned Pregnancy Intention) Among Participants Who Completed at Least Two Pregnancy Intention Questionnaires and Did Not Report a Pregnancy During Follow-up (N=8,449) **eTable 1.** Sociodemographic Factors of Participants Who Were Included in the Analysis Versus Those in the Entire Study Population, Nurses’ Health Study 3 **eTable 2.** Concordance Between Pregnancy Intention and Use of Contraceptives During Follow-up Among Women Who Reported Both Information on the Same Questionnaire, N=6,555 **eTable 3.** Sociodemographic Factors of Participants Who Did Not Get Pregnant During 12 Months of Follow up According to Change of Prospectively Reported Pregnancy Intention Status During Follow up **eTable 4.** Sociodemographic Factors of Participants Who Did Not Get Pregnant During 12 Months of Follow up According to Change of Prospectively Reported Pregnancy Intention Status During Follow up, Among Women Who Were Actively Trying at Baseline **eTable 5.** Sociodemographic Factors of Participants Who Did Not Get Pregnant During 12 Months of Follow up According to Change of Prospectively Reported Pregnancy Intention Status During Follow up, Among Women Who Were Contemplating Pregnancy at Baseline **eTable 6.** Sociodemographic Factors of Participants Who Did Not Get Pregnant During 12 Months of Follow up According to Change of Prospectively Reported Pregnancy Intention Status During Follow up, Among Women Who Were Not Trying at Baseline

Supplement 2.
Data Sharing Statement
