# Supplementary material for: Pregnancy Intention, Changes in Pregnancy Intention, and Pregnancy Incidence Among Female Nurses in North America
Source: JAMA Netw Open. 2023 May 3;6(5):e2311301. doi: 10.1001/jamanetworkopen.2023.11301 (PMC10157424; doi:10.1001/jamanetworkopen.2023.11301)
Supplement: Supplement 1. — eFigure 1. Flowchart of Cohort Design, Nurses’ Health Study 3 (NHS3), 2010–Present eFigure 2. Log-Normal Survival Function of the Duration of Ongoing Pregnancy Attempt (Solid Line) and Actual Distribution of Ongoing Pregnancy Attempt (Black Dots) Among Women Who Ever Reported Being Actively Trying to Get Pregnant, Nurses’ Health Study 3 Cohort (N = 5,249) eFigure 3. Kaplan-Meier Estimates of Cumulative Pregnancy Incidence Within 12 Months According to Prospectively Reported Preconception Pregnancy Intention at Baseline Among Women Aged 35 Years or Younger, Nurses’ Health Study 3 (2010–2022) eFigure 4. Kaplan-Meier Estimates of Cumulative Pregnancy Incidence Within 12 Months According to Prospectively Reported Preconception Pregnancy Intention at Baseline Among Partnered Women Aged 22 Years or Older, Did Not Report a History of Infertility, and Had Not Been Trying for Less Than 6 Months at Baseline, Nurses’ Health Study 3 (2010–2022) eFigure 5. Kaplan-Meier Estimates of Cumulative Pregnancy Incidence Within 12 Months According to Prospectively Reported Preconception Pregnancy Intention at Baseline, Restricting Follow up to Prior to the Pandemic, NHS3 (2010–2020) eFigure 6. Change in Pregnancy Intention Over 12 Months of Follow-up (Last Returned Pregnancy Intention) Among Participants Who Completed at Least Two Pregnancy Intention Questionnaires and Did Not Report a Pregnancy During Follow-up (N=8,449) eTable 1. Sociodemographic Factors of Participants Who Were Included in the Analysis Versus Those in the Entire Study Population, Nurses’ Health Study 3 eTable 2. Concordance Between Pregnancy Intention and Use of Contraceptives During Follow-up Among Women Who Reported Both Information on the Same Questionnaire, N=6,555 eTable 3. Sociodemographic Factors of Participants Who Did Not Get Pregnant During 12 Months of Follow up According to Change of Prospectively Reported Pregnancy Intention Status During Follow up eTable 4. Sociodemographic Factors of Participants Who Di [file jamanetwopen-e2311301-s001.pdf]

Supplementary Online Content

Wang S, Minguez-Alarcon L, Capotosto MP, et al. Pregnancy intention, changes in pregnancy intention, and pregnancy incidence among female nurses in North America. *JAMA Netw Open*. 2023;6(5):e2311301. doi:10.1001/jamanetworkopen.2023.11301

eFigure 1. Flowchart of Cohort Design, Nurses' Health Study 3 (NHS3), 2010–Present
eFigure 2. Log-Normal Survival Function of the Duration of Ongoing Pregnancy Attempt (Solid Line) and Actual Distribution of Ongoing Pregnancy Attempt (Black Dots) Among Women Who Ever Reported Being Actively Trying to Get Pregnant, Nurses' Health Study 3 Cohort (N=5,249)
eFigure 3. Kaplan-Meier Estimates of Cumulative Pregnancy Incidence Within 12 Months According to Prospectively Reported Preconception Pregnancy Intention at Baseline Among Women Aged 35 Years or Younger, Nurses' Health Study 3 (2010–2022)
eFigure 4. Kaplan-Meier Estimates of Cumulative Pregnancy Incidence Within 12 Months According to Prospectively Reported Preconception Pregnancy Intention at Baseline Among Partnered Women Aged 22 Years or Older, Did Not Report a History of Infertility, and Had Not Been Trying for Less Than 6 Months at Baseline, Nurses' Health Study 3 (2010–2022)
eFigure 5. Kaplan-Meier Estimates of Cumulative Pregnancy Incidence Within 12 Months According to Prospectively Reported Preconception Pregnancy Intention at Baseline, Restricting Follow up to Prior to the Pandemic, NHS3 (2010–2020)
eFigure 6. Change in Pregnancy Intention Over 12 Months of Follow-up (Last Returned Pregnancy Intention) Among Participants Who Completed at Least Two Pregnancy Intention Questionnaires and Did Not Report a Pregnancy During Follow-up (N=8,449)
eTable 1. Sociodemographic Factors of Participants Who Were Included in the Analysis Versus Those in the Entire Study Population, Nurses' Health Study 3
eTable 2. Concordance Between Pregnancy Intention and Use of Contraceptives During Follow-up Among Women Who Reported Both Information on the Same Questionnaire, N=6,555
eTable 3. Sociodemographic Factors of Participants Who Did Not Get Pregnant During 12 Months of Follow up According to Change of Prospectively Reported Pregnancy Intention Status During Follow up
eTable 4. Sociodemographic Factors of Participants Who Did Not Get Pregnant During 12 Months of Follow up According to Change of Prospectively Reported Pregnancy Intention Status During Follow up, Among Women Who Were Actively Trying at Baseline
eTable 5. Sociodemographic Factors of Participants Who Did Not Get Pregnant During 12 Months of Follow up According to Change of Prospectively Reported Pregnancy Intention Status During Follow up, Among Women Who Were Contemplating Pregnancy at Baseline
eTable 6. Sociodemographic Factors of Participants Who Did Not Get Pregnant During 12 Months of Follow up According to Change of Prospectively Reported Pregnancy Intention Status During Follow up, Among Women Who Were Not Trying at Baseline

This supplementary material has been provided by the authors to give readers additional information about their work.

© 2023 Wang S et al. *JAMA Network Open.*

eFigure 1. Flowchart of cohort design, Nurses' Health Study 3 (NHS3), 2010–present

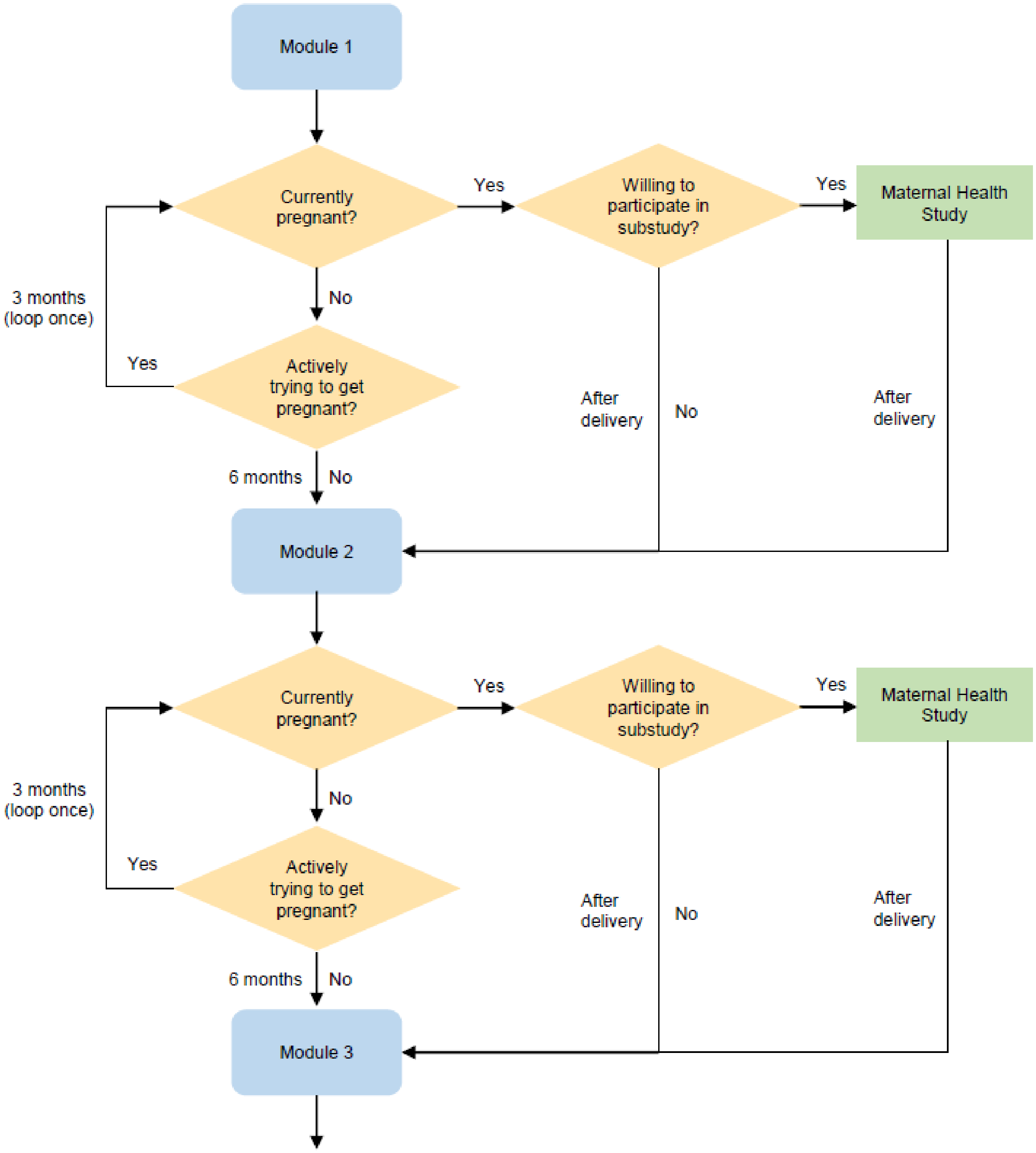

© 2023 Wang S et al. *JAMA Network Open.*

eFigure 2. Log-normal survival function of the duration of ongoing pregnancy attempt (solid line) and actual distribution of ongoing pregnancy attempt (black dots) among women who ever reported being actively trying to get pregnant, Nurses' Health Study 3 cohort (N=5,249)

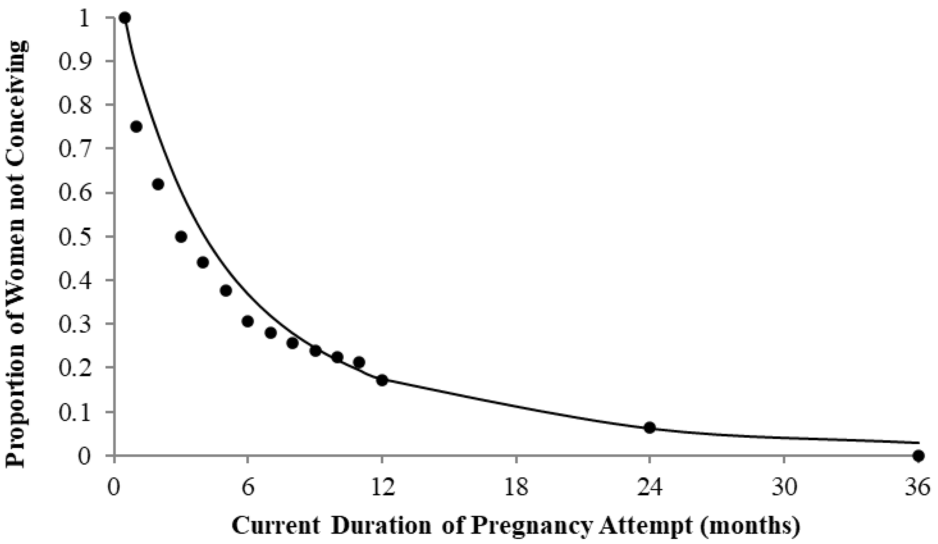

© 2023 Wang S et al. *JAMA Network Open.*

eFigure 3. Kaplan-Meier estimates of cumulative pregnancy incidence within 12 months according to prospectively reported preconception pregnancy intention at baseline among women aged 35 years or younger, Nurses' Health Study 3 (2010–2022)

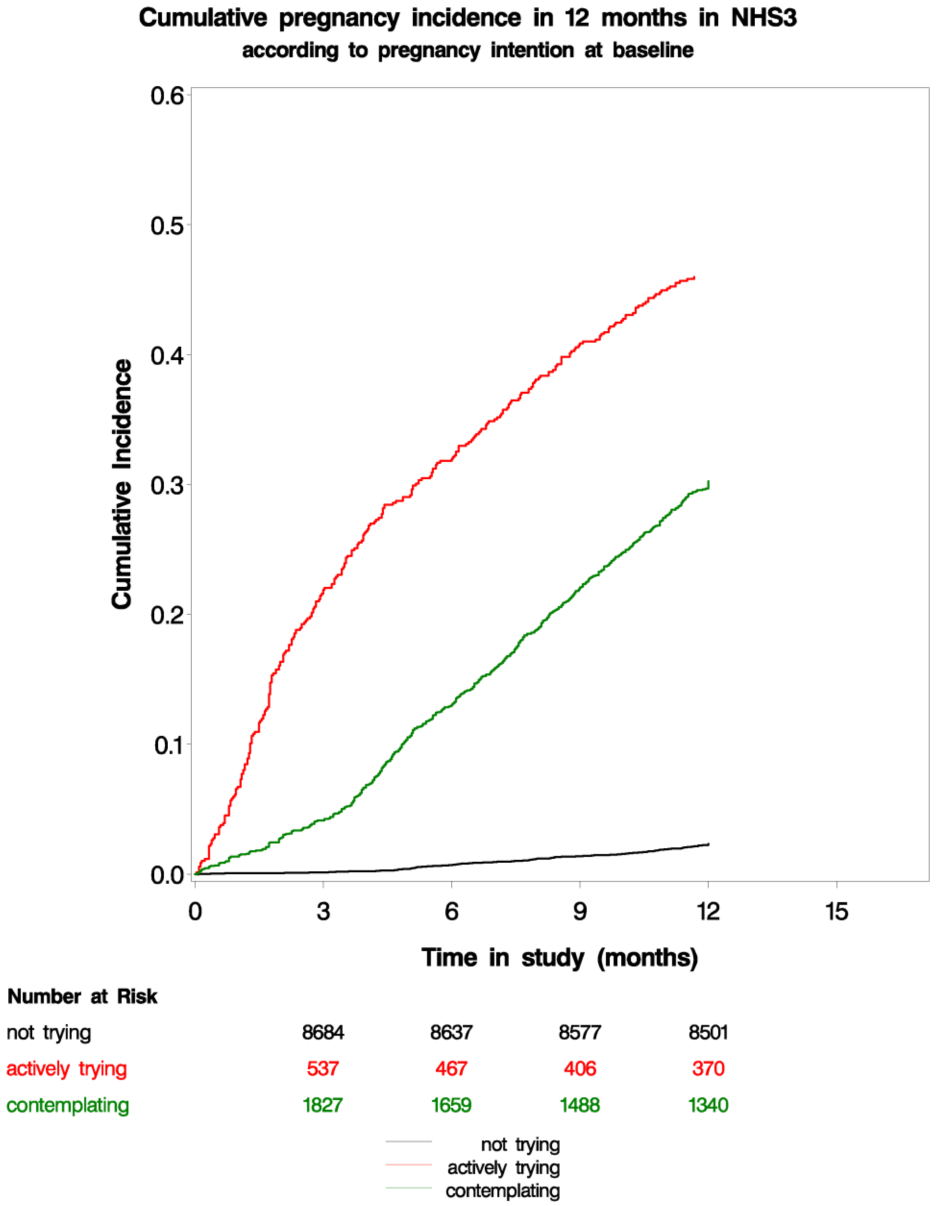

P log-rank<0.001

© 2023 Wang S et al. *JAMA Network Open.*

eFigure 4. Kaplan-Meier estimates of cumulative pregnancy incidence within 12 months according to prospectively reported preconception pregnancy intention at baseline among partnered women ages 22 years or older**, did not report a history of infertility, and had not been trying for less than 6 months at baseline, Nurses' Health Study 3 (2010–2022)**

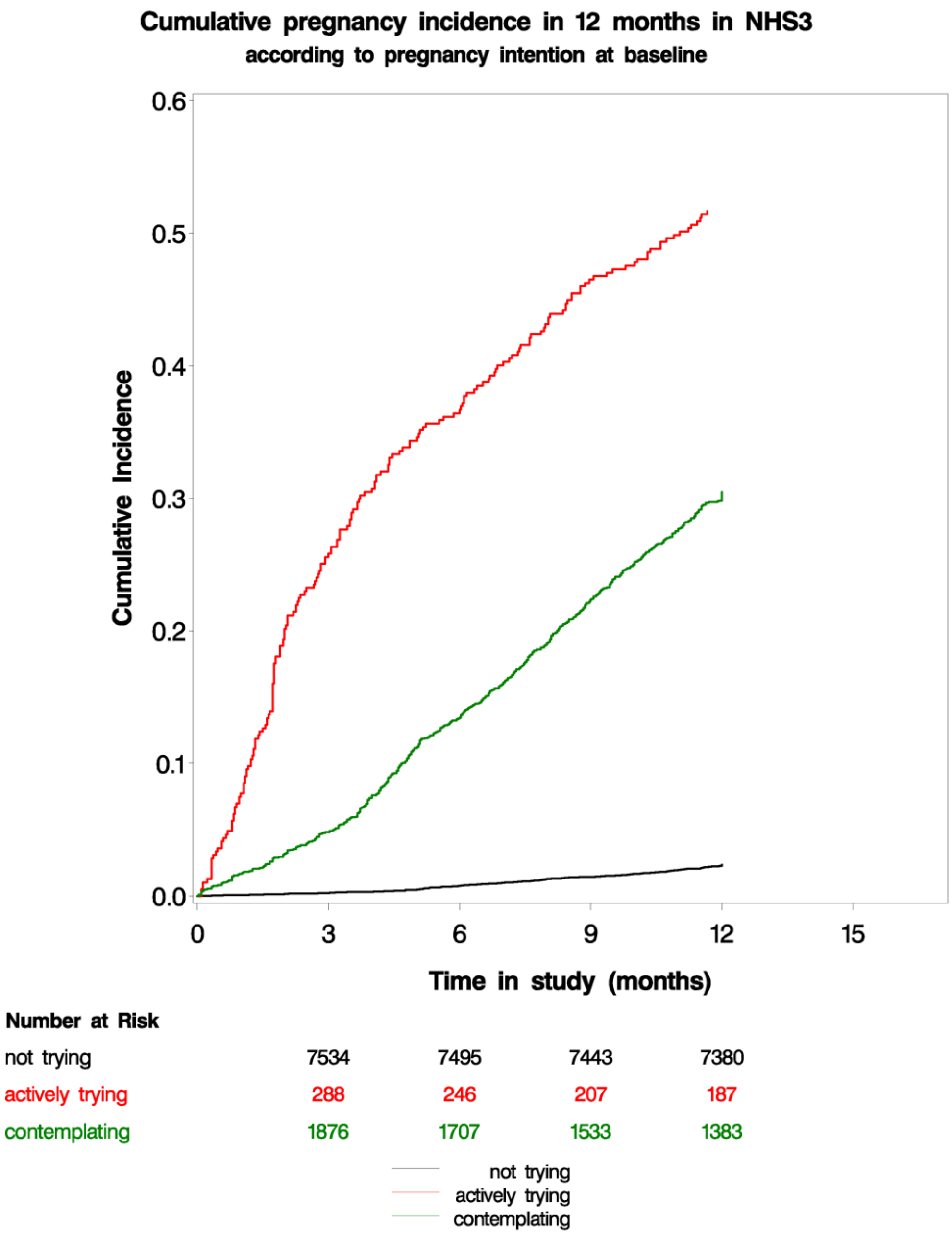

P log-rank<0.001

© 2023 Wang S et al. *JAMA Network Open.*

eFigure 5. Kaplan-Meier estimates of cumulative pregnancy incidence within 12 months according to prospectively reported preconception pregnancy intention at baseline, restricting follow up to prior to the pandemic, NHS3 (2010–2020)

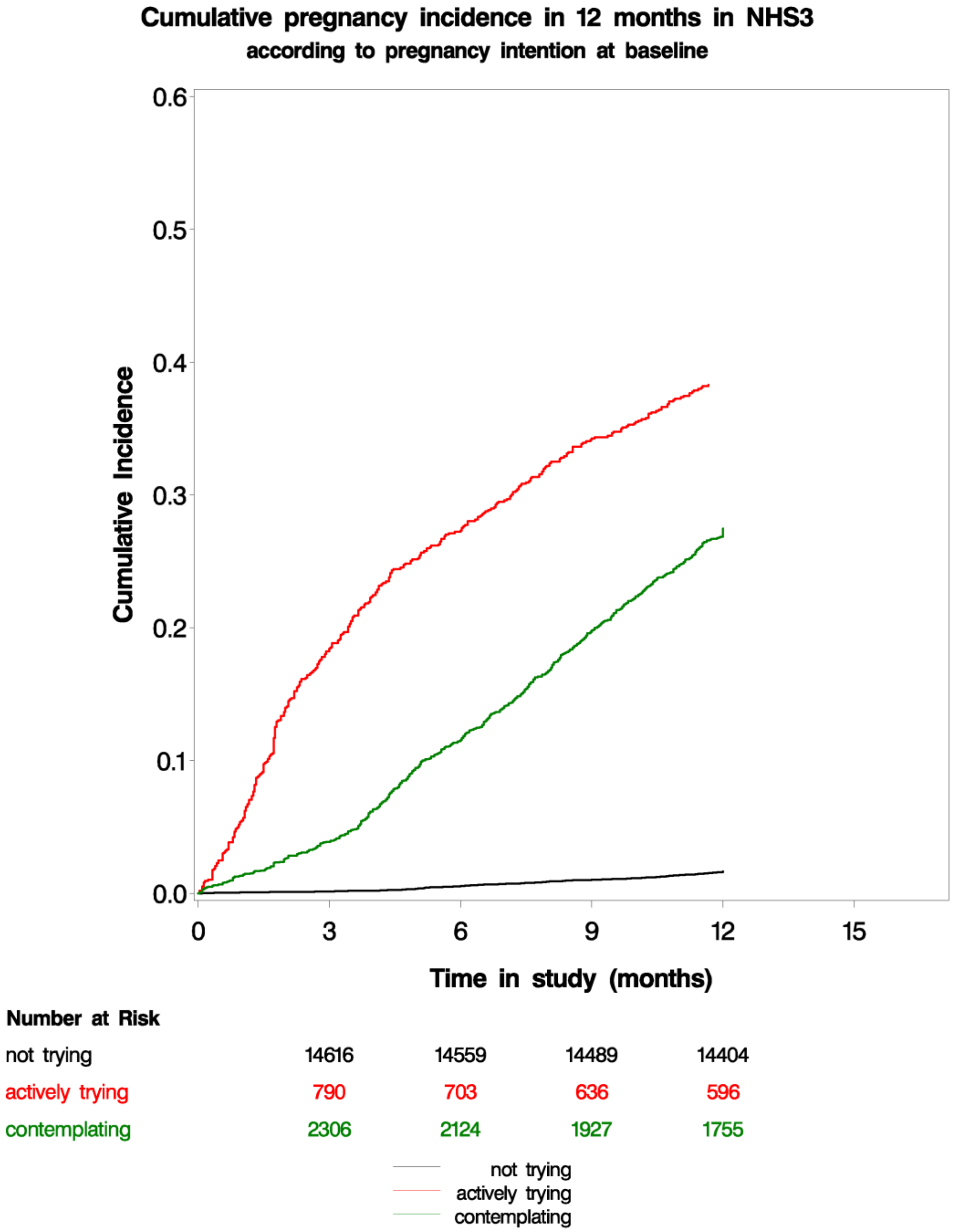

P log-rank<0.001

© 2023 Wang S et al. *JAMA Network Open.*

eFigure 6. Change in pregnancy intention over 12 months of follow-up (last returned pregnancy intention) among participants who completed at least two pregnancy intention questionnaires and did not report a pregnancy during follow-up (N=8,449)

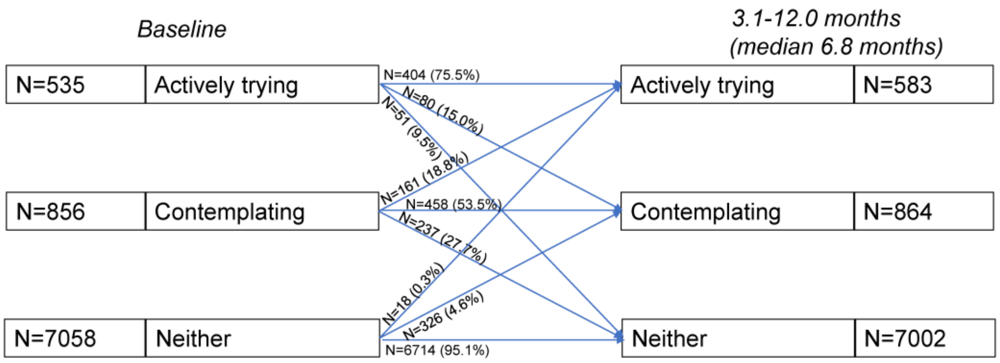

© 2023 Wang S et al. *JAMA Network Open.*

eTable 1. Sociodemographic factors of participants who were included in the analysis versus those in the entire study population, Nurses' Health Study 3

	Study population	Participants not included in the current study	All Nurses' Health Study 3 participants
n (%)	N=18376	N=30324	N=48700
Age, mean (SD), years	32.4 (6.5)	34.6 (7.9)	33.8 (7.5)
Race/ethnicity			
non-Hispanic White	16171 (88.0)	25116 (82.8)	41287 (84.8)
Hispanic	765 (4.2)	1766 (5.8)	2531 (5.2)
Others	1440 (7.8)	3442 (11.4)	4882 (10.0)
Marital Status			
Never married	6571 (35.8)	8444 (27.9)	15015 (30.8)
Married or domestic partnership	10522 (57.3)	18611 (61.4)	29133 (59.8)
Separated or divorced	1212 (6.6)	3037 (10.0)	4249 (8.7)
Widowed	28 (0.2)	98 (0.3)	126 (0.3)
Nulliparous	10521 (57.3)	15664 (51.7)	26185 (53.8)
Education attainment			
Diploma in Nursing	598 (3.3)	2019 (6.7)	2627 (5.4)
Associate's Degree	9625 (52.4)	14010 (46.2)	23635 (48.5)
Bachelors	3380 (18.4)	4812 (15.9)	8192 (16.8)
Master's or Doctorate	277 (1.5)	379 (1.2)	656 (1.4)
Partner's education[a]			
High school or lower	3998 (38.0)	8519 (45.8)	12517 (43.0)
College	4026 (38.3)	6225 (35.1)	10551 (36.2)
Grad school	2488 (23.7)	3518 (18.9)	6006 (20.6)
Region			
West	3610 (19.7)	5420 (17.9)	9030 (18.5)
Midwest	5238 (28.5)	8194 (27.0)	13432 (27.6)
South	4112 (22.4)	7958 (26.2)	12070 (24.8)
Northeast	4463 (24.3)	6771 (22.3)	11234 (23.1)
Military or outside US	953 (5.2)	1981 (6.5)	2934 (6.2)
	N=11361	N=8438	N=19799
Household income			
<50k	1184 (10.4)	814 (9.7)	1998 (10.1)
50-100k	3231 (28.4)	2351 (27.9)	5582 (28.2)
100k-200k	5209 (45.9)	3818 (45.3)	9027 (45.6)
>200k	1332 (11.7)	1023 (12.1)	2355 (11.9)
	N=10470	N=6494	N=16964
Attendance to religious services			
More than once a week	2105 (20.1)	1408 (21.7)	3513 (20.7)
Private religious activity			
More than once a week	3578 (34.2)	2456 (37.8)	6034 (35.6)

Numbers might not add up to 100% because of missingness

[a]Unpartnered women were not included

© 2023 Wang S et al. *JAMA Network Open.*

eTable 2. Concordance between pregnancy intention and use of contraceptives during follow-up among women who reported both information on the same questionnaire, N=6,555

			Pregnancy intention		
			Actively trying	Contemplating	Not trying nor think will become pregnant
n (%)			N=342	N=729	N=5484
Currently using contraception	Yes		22 (6.4)	500 (68.6)	4037 (73.6)
		Oral contraceptives	3 (0.9)	164 (22.5)	1331 (24.3)
		Condom	6 (1.8)	159 (21.8)	664 (12.1)
		IUD	2 (0.6)	69 (9.5)	750 (13.7)
		Vasectomy	0 (0)	1 (0.1)	676 (12.3)
		Patch	0 (0)	34 (4.7)	199 (3.6)
		Tubal ligation	0 (0)	0 (0)	185 (3.4)
		Rhythm	4 (1.2)	41 (5.6)	69 (1.3)
		Natural family planning	6 (1.8)	22 (3.0)	48 (0.9)
		Implanon	0 (0)	1 (0.1)	45 (0.8)
		Depo	1 (0.3)	2 (0.3)	22 (0.4)
		Diaphragm/ cervical cap	0 (0)	5 (0.7)	17 (0.3)
		Foam	0 (0)	1 (0.1)	20 (0.4)
		Other hormone	0 (0)	1 (0.1)	11 (0.2)
	No		320 (93.6)	229 (31.4)	1447 (26.4)

© 2023 Wang S et al. *JAMA Network Open.*

eTable 3. Sociodemographic factors of participants who did not get pregnant during 12 months of follow up according to change of prospectively reported pregnancy intention status during follow up

	Change of pregnancy intention	
	No	Yes
n (%)	N=7576	N=873
Age, mean (SD), years	31.9 (6.9)	31.5 (4.9)
Age groups, years		
19–24	1313 (17.3)	54 (6.2)
25–34	3383 (44.7)	585 (67.0)
35–44	2880 (38.0)	234 (26.8)
Race/ethnicity		
non-Hispanic White	6606 (87.2)	739 (84.7)
Hispanic	350 (4.6)	50 (5.7)
All other minorities[a]	620 (8.2)	84 (9.6)
Marital Status		
Never married	3345 (44.2)	202 (23.1)
Married or domestic partnership	3683 (48.6)	634 (72.6)
Separated or divorced	517 (6.8)	36 (4.1)
Widowed	10 (0.1)	0 (0)
Nulliparous	4669 (61.6)	592 (67.8)
Education attainment		
Diploma in Nursing	502 (6.6)	52 (6.0)
Associate's Degree	3788 (50.0)	464 (53.2)
Bachelors	1134 (15.0)	179 (20.5)
Master's or Doctorate	98 (1.3)	16 (1.8)
Partner's education[b]		
High school or lower	1458 (39.6)	226 (35.7)
College	1400 (38.0)	243 (38.4)
Grad school	823 (22.4)	165 (26.0)
Region		
West	1498 (19.8)	178 (20.4)
Midwest	2186 (28.9)	235 (26.9)
South	1596 (21.1)	198 (22.7)
Northeast	1821 (24.0)	197 (22.6)
Military or outside US	475 (6.3)	65 (7.5)
	N=4503	N=516
Household income		
<50k	711 (15.8)	41 (8.0)
50–100k	1412 (31.4)	156 (30.2)
100k–200k	1822 (40.5)	237 (45.9)
>200k	384 (8.5)	63 (12.2)
	N=3958	N=483
Attendance to religious services		
More than once a week	737 (18.6)	97 (20.1)
Private religious activity		
More than once a week	1234 (31.2)	171 (35.4)

Numbers might not add up to 100% because of missingness

[a]African American/Black, American Indian/Alaska Native, Asian or Native Hawaiian or Pacific Islander

[b]Unpartnered women were not included

© 2023 Wang S et al. *JAMA Network Open.*

eTable 4. Sociodemographic factors of participants who did not get pregnant during 12 months of follow up according to change of prospectively reported pregnancy intention status during follow up, among women who were actively trying at baseline

	Change of pregnancy intention	
	No	Changed away from trying
n (%)	N=404	N=131
Age, mean (SD), years	32.9 (4.7)	34.5 (4.9)
Age groups, years		
19–24	11 (2.7)	2 (1.5)
25–34	247 (61.1)	62 (47.3)
35–44	146 (36.1)	67 (51.2)
Race/ethnicity		
non-Hispanic White	354 (87.6)	98 (74.8)
Hispanic	19 (4.7)	14 (10.7)
All other minorities[a]	31 (7.7)	19 (14.5)
Marital Status		
Never married	19 (4.7)	17 (13.0)
Married or domestic partnership	368 (91.1)	112 (85.5)
Separated or divorced	16 (4.0)	2 (1.5)
Widowed	1 (0.3)	0 (0)
Nulliparous	278 (68.8)	87 (66.4)
Education attainment		
Diploma in Nursing	17 (4.2)	5 (3.8)
Associate's Degree	191 (47.3)	56 (42.8)
Bachelors	93 (23.0)	37 (28.2)
Master's or Doctorate	5 (1.2)	4 (3.1)
Partner's education[a]		
High school or lower	145 (39.4)	52 (46.4)
College	131 (35.6)	28 (25.0)
Grad school	92 (25.0)	32 (28.6)
Region		
West	86 (21.3)	28 (21.4)
Midwest	122 (30.2)	37 (28.2)
South	93 (23.0)	35 (26.7)
Northeast	86 (21.3)	21 (16.0)
Military or outside US	17 (4.2)	10 (7.6)
	N=219	N=86
Household income		
<50k	8 (3.7)	4 (4.7)
50–100k	51 (23.3)	29 (33.7)
100k–200k	119 (54.3)	43 (50.0)
>200k	29 (13.2)	6 (7.0)
	N=194	N=77
Attendance to religious services		
More than once a week	48 (24.7)	18 (23.4)
Private religious activity		
More than once a week	70 (36.1)	30 (39.0)

Numbers might not add up to 100% because of missingness
[a]African American/Black, American Indian/Alaska Native, Asian or Native Hawaiian or Pacific Islander
[b]Unpartnered women were not included

© 2023 Wang S et al. *JAMA Network Open.*

eTable 5. Sociodemographic factors of participants who did not get pregnant during 12 months of follow up according to change of prospectively reported pregnancy intention status during follow up, among women who were contemplating pregnancy at baseline

	Change of pregnancy intention			
	No	Yes		
		Changed toward trying	Changed away from trying	Any change
n (%)	N=458	N=161	N=237	N=398
Age, mean (SD), years	31.4 (4.7)	31.3 (4.4)	31.6 (5.1)	31.5 (4.8)
Age groups, years				
19–24	27 (5.9)	3 (1.9)	18 (7.6)	21 (5.3)
25–34	317 (69.2)	120 (74.5)	152 (64.1)	272 (68.3)
35–44	114 (24.9)	38 (23.6)	67 (28.3)	105 (26.4)
Race/ethnicity				
non-Hispanic White	397 (86.7)	138 (85.7)	209 (88.2)	347 (87.2)
Hispanic	19 (4.2)	7 (4.4)	9 (3.8)	16 (4.0)
All other minorities[a]	42 (9.2)	16 (9.9)	19 (8.0)	35 (8.8)
Marital Status				
Never married	97 (21.2)	18 (11.2)	53 (22.4)	71 (17.8)
Married or domestic partnership	341 (74.5)	136 (84.5)	173 (73.0)	309 (77.6)
Separated or divorced	20 (4.4)	7 (4.4)	11 (4.6)	18 (4.5)
Widowed	0 (0)	0 (0)	0 (0)	0 (0)
Nulliparous	303 (66.2)	110 (68.3)	151 (63.7)	261 (65.6)
Education attainment				
Diploma in Nursing	26 (5.7)	11 (6.8)	9 (3.8)	20 (5.0)
Associate's Degree	237 (51.8)	91 (56.5)	132 (55.7)	223 (56.0)
Bachelors	86 (18.8)	35 (21.7)	45 (19.0)	80 (20.1)
Master’s or Doctorate	5 (1.1)	2 (1.2)	4 (1.7)	6 (1.5)
Partner’s education[b]				
High school or lower	106 (31.1)	42 (30.9)	65 (37.6)	107 (34.6)
College	147 (43.1)	70 (51.5)	63 (36.4)	133 (43.0)
Grad school	88 (25.8)	24 (17.7)	45 (26.0)	69 (22.3)
Region				
West	107 (23.4)	29 (18.0)	44 (18.6)	73 (18.3)
Midwest	123 (26.9)	43 (26.7)	58 (24.8)	101 (25.4)
South	100 (12.8)	37 (23.0)	58 (24.5)	95 (23.9)
Northeast	101 (22.1)	41 (25.5)	61 (25.7)	102 (25.6)
Military or outside US	27 (5.9)	11 (6.8)	16 (6.8)	27 (6.8)
	N=266	N=98	N=128	N=226
Household income				
<50k	19 (7.1)	3 (3.1)	9 (7.0)	12 (5.3)
50–100k	76 (28.6)	30 (30.6)	35 (27.3)	65 (28.8)
100k–200k	139 (52.3)	48 (49.0)	62 (48.4)	110 (48.7)
>200k	26 (9.8)	11 (11.2)	16 (12.5)	27 (12.0)
	N=225	N=96	N=115	N=211
Attendance to religious services				
More than once a week	36 (16.0)	13 (13.5)	25 (21.7)	38 (18.0)
Private religious activity				
More than once a week	69 (30.7)	28 (29.2)	43 (37.4)	71 (33.7)

Numbers might not add up to 100% because of missingness
[a]African American/Black, American Indian/Alaska Native, Asian or Native Hawaiian or Pacific Islander
[b]Unpartnered women were not included

© 2023 Wang S et al. *JAMA Network Open.*

eTable 6. Sociodemographic factors of participants who did not get pregnant during 12 months of follow up according to change of prospectively reported pregnancy intention status during follow up, among women who were not trying at baseline

	Change of pregnancy intention	
	No	Changed toward trying
n (%)	N=6714	N=344
Age, mean (SD), years	31.9 (7.1)	30.6 (4.7)
Age groups, years		
19–24	1275 (19.0)	31 (9.0)
25–34	2819 (42.0)	251 (73.0)
35–44	2620 (39.0)	62 (18.0)
Race/ethnicity		
non-Hispanic White	5855 (87.2)	294 (84.5)
Hispanic	312 (4.7)	20 (5.8)
All other minorities[a]	547 (8.2)	30 (8.7)
Marital Status		
Never married	3229 (48.1)	114 (33.1)
Married or domestic partnership	2974 (44.3)	213 (61.9)
Separated or divorced	481 (7.2)	16 (4.7)
Widowed	10 (0.2)	0 (0)
Nulliparous	2626 (39.1)	100 (29.1)
Education attainment		
Diploma in Nursing	459 (6.8)	27 (7.9)
Associate's Degree	3360 (50.0)	185 (53.8)
Bachelors	955 (14.2)	62 (18.0)
Master's or Doctorate	88 (1.3)	6 (1.7)
Partner's education[b]		
High school or lower	1207 (40.6)	67 (31.5)
College	1122 (37.7)	82 (38.5)
Grad school	643 (21.6)	64 (30.1)
Region		
West	1305 (19.4)	77 (22.4)
Midwest	1941 (28.9)	97 (28.2)
South	1403 (20.9)	68 (19.8)
Northeast	1634 (24.3)	74 (21.5)
Military or outside US	431 (6.4)	28 (8.1)
	N=4018	N=204
Household income		
<50k	684 (17.0)	25 (12.3)
50–100k	1285 (32.0)	62 (30.4)
100k–200k	1564 (38.9)	84 (41.2)
>200k	329 (8.2)	30 (14.7)
Attendance to religious services		
More than once a week	653 (18.5)	41 (21.0)
Private religious activity		
More than once a week	1095 (30.9)	70 (35.9)

Numbers might not add up to 100% because of missingness

[a]African American/Black, American Indian/Alaska Native, Asian or Native Hawaiian or Pacific Islander

[b]Unpartnered women were not included

© 2023 Wang S et al. *JAMA Network Open.*
