# Supplementary material for: Pregnancy Intention, Changes in Pregnancy Intention, and Pregnancy Incidence Among Female Nurses in North America
Source: JAMA Netw Open. 2023 May 3;6(5):e2311301. doi: 10.1001/jamanetworkopen.2023.11301 (PMC10157424; doi:10.1001/jamanetworkopen.2023.11301)
Supplement: Supplement 2. — Data Sharing Statement [file jamanetwopen-e2311301-s002.pdf]

Data Sharing Statement

Wang. Pregnancy Intention, Changes in Pregnancy Intention, and Pregnancy Incidence Among Female Nurses in North America. *JAMA Netw Open*. Published May 03, 2023. doi:10.1001/jamanetworkopen.2023.11301

Data
Data available: No

Additional Information
Explanation for why data not available: Further information including the procedures to obtain and access data from the Nurses' Health Studies is described at https://www.nurseshealthstudy.org/researchers (contact email: nhsaccess@channing.harvard.edu).
